# Type 2 diabetes mellitus as a state of altered drug pharmacokinetic-pharmacodynamic parameters: an update on recent developments and clinical implications

**DOI:** 10.1177/20420188241305021

**Published:** 2026-04-02

**Authors:** Ntethelelo Sibiya, Chiamaka Onyekwuluje, Auxiliare Kuretu, Omobonlale Ayodele, Nokwanda Ngcobo, Phikelelani Ngubane, Andile Khathi

**Affiliations:** Pharmacology Division, Faculty of Pharmacy, Rhodes University, Room 405, 4th Floor, Artillery Road, Makhanda 6139, South Africa; Pharmacology Division, Faculty of Pharmacy, Rhodes University, Makhanda, South Africa; Pharmacology Division, Faculty of Pharmacy, Rhodes University, Makhanda, South Africa; Pharmacology Division, Faculty of Pharmacy, Rhodes University, Makhanda, South Africa; School of Health Sciences, College of Health Sciences, University of KwaZulu-Natal, Durban, South Africa; School of Laboratory Medicine and Medical Sciences, College of Health Sciences, University of KwaZulu-Natal, Durban, South Africa; School of Laboratory Medicine and Medical Sciences, College of Health Sciences, University of KwaZulu-Natal, Durban, South Africa

**Keywords:** CYP450, diabetes mellitus, diabetic nephropathy, gastric emptying, inflammation hypoalbuminemia, intestinal drug transporters, pharmacokinetic-pharmacodynamic behaviour

## Abstract

Type 2 diabetes mellitus (T2DM) is a chronic metabolic disorder characterised by hyperglycaemia. And its global prevalence is increasing at an alarming rate. Uncontrolled diabetes can progress to an alteration of tissue and organ system morphology and function, resulting in complications that affect drug pharmacokinetics (PKs) and pharmacodynamics (PDs). Diabetes is associated with several physiological changes including delayed gastric emptying, intestinal drug transporters, cytochrome P450, hypoalbuminemia and an increase or decline in glomerular filtration rate. From a PK point of view, these pathophysiological changes in 2 diabetes mellitus could lead to altered drug absorption, distribution, metabolism and clearance. Consequently, such PKs changes can lead to variability in the PD responses of many drugs, increasing the risk of treatment failure or adverse effects. Furthermore, T2DM sensitise individuals to co-morbidities, thus, necessitating polypharmacy, heightening the risk of drug-drug interactions. A diabetic state proves to be a fertile ground for PK-PD profile variability for several agents including antidiabetics, antibiotics, antifungals and central nervous system drugs. Such variability may necessitate careful patient monitoring. In many cases, dose adjustment or drug switching may be required to optimise therapeutic outcomes. Given the rising incidence and early onset of diabetes, it is increasingly important to understand the unique PK-PD profiles of drugs in this population. For these reasons, research into the PK-PD behaviour of drugs should be accelerated. Through heightened understanding, we envisage consensus and clear guidelines for the management of various conditions in a diabetic state would be sought.

## Introduction

Type 2 diabetes mellitus (T2DM) is a disease primarily characterised by hyperglycaemia, resulting from either a lack of insulin production or reduced insulin sensitivity in key target tissues such as skeletal muscle, adipose and hepatic tissues.^1^ The disease is associated with persistent low-grade inflammation and oxidative stress, which can precipitate the onset and progression of diabetes complications such as cardiovascular, retinopathy, nephropathy and neuropathy.^2^ Despite efforts to advance our understanding and develop preventative strategies, the global trajectory of diabetes continues to rise. In addition, many cases of diabetes remain undiagnosed. Whilst there is currently no cure for diabetes, some reports suggest that remission is possible through lifestyle modifications, including dietary interventions, particularly for T2DM.^3^ Central to the management of T2DM, is strict glycaemic control, achieved through various antihyperglycaemic agents, such as biguanides, thiazolidinediones (TZDs), sulfonylureas, DPP-IV inhibitors and SGLT-2 inhibitors. In cases where oral anti-hyperglycaemic agents are insufficient to achieve glucose control, Insulin therapy is recommended as an add-on therapy.^4^ Poor glycaemic control often leads to long-term complications, such as diabetic nephropathy (DN), neuropathy, retinopathy and cardiovascular and hepatic complications.^5^ From a pathophysiological perspective, the underlying mechanisms of diabetes-related complications include increased oxidative stress, low-grade inflammation, advanced glycation end products (AGEs) and activation of the polyol and hexosamine pathways.^6^

Diabetic patients are also more susceptible to several other diseases, including cancer, bacterial, viral and fungal infections, due to compromised immune function.^7^ As a result, polypharmacy is often inevitable for many diabetic patients. Recent evidence further suggests that this metabolic condition can affect the metabolism of pharmacological agents, leading to altered pharmacokinetic-pharmacodynamic (PK-PD) behaviour.^8^ Consequently, drug-drug interactions (DDIs) in diabetic patients may alter the PK-PD profile of certain pharmacological agents, thus, impacting therapeutic efficacy.^9^ Furthermore, the pathophysiological changes associated with diabetes can directly affect the PK-PD behaviour of certain therapeutic agents. Since drug metabolism and elimination are primarily achieved through hepatic biotransformation and renal clearance, impaired hepatic and renal function in diabetic patients are often linked to poor drug handling, potentially compromising therapeutic outcomes.^10^ This presents a double-edged sword, where the heightened risk of co-morbidities in diabetes is compounded by the likelihood of pharmacotherapeutic failure or adverse effects due to altered PK-PD profiles.

In this review, we aim to provide an overview of recent developments in the relationship between diabetes and the altered PK-PD behaviour of certain pharmacological agents. We focus on four key aspects in the context of a diabetic state: gastrointestinal changes, cytochrome P450 (CYP450) modulation, alterations in plasma albumin concentrations and changes in glomerular filtration rate (GFR). These changes are associated with reduced predictability of PK-PD properties in diabetic patients. We envisage this review will help consolidate management strategies for other disorders in diabetic patients, considering the altered PK-PD profiles of certain drugs resulting from diabetes.

## Gastrointestinal tract functional changes in diabetes

The GIT plays a vital role in functions such as food digestion, nutrient absorption, water and enzyme secretion and waste elimination. These functions are regulated by a complex neural network known as the enteric nervous system (ENS).^11^ The ENS spans the entire GIT and communicates with the central nervous system (CNS) through sensory neurons that transmit visceral sensations, including pain and fullness. In addition, the ENS connects to the CNS via efferent sympathetic and parasympathetic pathways, which regulate motility, secretion and circulation within the GIT.^12^ Numerous animal studies have shown that diabetes can alter chemical coding. Different subpopulations of enteric neurons exhibit varied responses to diabetes; some degenerate, others experience changes in neurotransmitter content without degeneration, and some remain unaffected.^13^ Interestingly, even neurons containing the same neurotransmitter can be affected differently depending on the specific region of the GIT they innervate.^14^ This neuronal remodelling alters the balance between inhibitory and contractile neurons, disrupting nerve-mediated muscle function, which may contribute to the motility issues commonly observed in diabetes.^13,15^

The impact of diabetes on small intestine and colonic functions has not been as thoroughly studied. However, both constipation and diarrhoea are reported more frequently in diabetic patients.^15^ Early research suggested that diabetes slows intestinal transit time in animal models, which could lead to bacterial overgrowth and subsequent diarrhoea.^15^ However, later studies found that some models exhibited accelerated intestinal transit. This faster transit was attributed to autonomic neuropathy and diabetes-induced denervation of sympathetic nerve terminals.^13,15^ In contrast, colonic transit time is often prolonged, and constipation is a common symptom in diabetic patients.^15,16^

Numerous studies have reported a loss of neuronal nitric oxide synthase (nNOS) neurons in diabetes, with impaired nitrergic function in the stomach leading to delayed gastric emptying or altered accommodation.^17,18^ Chandrasekharan and Srinivasan have reported on numerous studies where in both streptozotocin (STZ)-mice and non-obese diabetic mice, insulin therapy has been shown to restore nitrergic function in the stomach.^19^ Similarly, a reduction in nNOS expression was observed in the antral myenteric plexus of STZ-diabetic rats after 3 months and in spontaneously diabetic BB/W rats after 6 months of diabetes. In addition, a loss of ileal myenteric neurons stained for NADPH diaphorase was detected at 8 weeks of diabetes in mice.^19^ During the early stages of diabetes, nNOS content and function decline, but as the disease progresses, nitrergic degeneration occurs, resulting in a complete loss of nitrergic function. Jejunal sections from diabetic patients revealed a decrease in both nNOS neurons and interstitial cells of Cajal (ICC).^20,21^ A similar reduction in ICC and nNOS neurons was noted in T2DM patients, with the severity of the condition correlating to the degree of loss compared to controls.^19–21^ Interestingly, the susceptibility of nitrergic neurons in the GIT appears to vary by region, with the stomach and proximal intestine being affected first, whilst the distal colon is impacted later.^22^ Within the stomach, neurons along the lesser curvature are more vulnerable than those along the greater curvature. The loss of nNOS neurons may be due to increased apoptosis.^13,15,23^

In the later stages of diabetes, there seems to be an increase in nitrergic neurons, likely as a regenerative response to the early loss of nNOS neurons.^24^ Studies have noted an increase in nNOS levels in the myenteric plexi of the duodenum and ileum at 32 weeks of diabetes. A subset of nNOS neurons lacking heme oxygenase 2 has been found to be more vulnerable to diabetic changes.^13,25^ Overall, whilst diabetes is generally associated with a reduction in nNOS neurons, there may be some regeneration at later stages. These alterations contribute to the motility disturbances commonly seen in diabetes.

Thus, disease states such as T2DM can lead to the loss of enteric neurons, as well as neuronal and smooth muscle dysfunction, causing subsequent gastrointestinal (GI) complications. When chronic hyperglycaemia leads to changes in intracellular glucose metabolism, resulting in the formation of AGEs, osmotic and oxidative stress and inflammation. These factors collectively lead to cellular damage, ultimately leading to glucose neurotoxicity, which can alter gastric emptying and drug transporters in the intestine.

### Disturbances in gastric emptying

GI motility is affected by the structural remodelling of oesophageal musculature and smooth muscle fibre innervation due to diabetic neuropathy. Hyperglycaemia has been shown to exacerbate abnormally heightened pyloric contractility, leading to decreased antral pressure waves, reduced antral motor activity and increased pyloric pressure waves. As a result, patients often experience distressing symptoms such as early satiety, bloating, nausea and vomiting, which can lead to spontaneous contractions and gastroparesis.^19,26^ Diabetic gastroparesis is characterised by the stomach’s inability to move food into the intestines, resulting in common symptoms like bloating, nausea and heartburn. Diabetes is the second leading cause of gastroparesis, with 5.4 million diabetic patients affected by this condition. Diabetic gastroparesis often leads to pill burden, which can result in compliance and/or adherence issues.^27^ The condition is particularly challenging due to its unpredictable nature, with symptoms varying from patient to patient and lacking consistent patterns. This unpredictability complicates diagnosis and makes glycaemic management difficult. In extreme cases, vomiting can lead to medication loss, making it harder to achieve therapeutic doses and complicating pharmacotherapeutic management. From an antimicrobial point of view, this represents yet another challenge, as suboptimal doses could precipitate antimicrobial resistance. Therefore, it is paramount to prioritise the diagnosis and management of diabetic gastroparesis, as it could serve as a gateway for the successful management of other co-morbidities, especially where oral drug administration is sought.

Management of diabetic gastroparesis involves various approaches, including lifestyle modifications, glycaemic control, pharmacological treatments and surgical interventions in refractory cases.^27^ Currently, metoclopramide is the only drug approved by the Food and Drug Administration for the treatment of diabetic gastroparesis. Diabetic gastroparesis may also limit the oral administration of drugs, necessitating the exploration of alternative administration routes. What we have highlighted above is paramount for clinicians to consider for diabetic patients when managing comorbidity.

### Altered intestinal drug transport

A series of transporters, such as P-glycoprotein (P-gp), and monocarboxylate transporter 6 (MCT6), as well as enzymes such as CYP450s, are expressed in enterocytes. These transporters regulate intestinal absorption and distribution, and first-hepatic metabolism.^28^ P-gp serves as an exclusion barrier for several xenobiotics and drugs, hence, its expression and activity regulate the quantities of drugs entering the circulation. Several studies have shown that diabetes diminishes the expression and efficacy of intestinal P-gp, leading to increased intestinal absorption and heightened plasma exposure after oral administration of P-gp substrates. For instance, clinical research has demonstrated higher serum concentrations of digoxin in diabetic individuals compared to non-diabetic individuals following an oral dose of digoxin.^29,30^ This is particularly concerning given digoxin’s narrow therapeutic index, which increases the risk of digoxin-induced toxicity, including potentially fatal cardiovascular complications. Furthermore, in diabetic animal models, such as STZ-induced diabetic rats, P-gp upregulation has been observed, along with a concomitant reduction in the plasma concentrations of P-gp substrates such as verapamil and atorvastatin. This poses a challenge in achieving optimal plasma concentrations for these drugs. MCT6, located on the apical side of human intestinal villous epithelial cells, facilitates the intestinal absorption of drugs such as nateglinide and loop diuretics such as furosemide and bumetanide. In diabetic patients, a significant decrease in plasma exposure to furosemide has been attributed to the downregulation of this transporter due to T2DM complications.^31^

## Hepatic CYP450 enzymes

The CYP450 system consists of a family of enzymes responsible for metabolising numerous medications.^32^ Whilst primarily found in hepatic tissue, these enzymes are also present in extra-hepatic tissues such as the small intestine, lungs, kidneys and heart.^33,34^ In the liver, they play a key role in first-pass metabolism, which explains the higher PK variability observed with oral drugs compared to those administered intravenously.^33,35^ The most extensively studied CYP450 isoforms include CYP1A2, CYP2C9, CYP2C19, CYP2D6 and CYP3A4/5, which together metabolise over 90% of substrate drugs, significantly influencing their PK-PD behaviour.^33^

Drug metabolism in the liver involves three major steps: hepatic uptake (transporter-mediated), phase I reactions and phase II metabolic reactions. In phase I reactions, CYP450 enzymes oxidise, reduce or hydrolyse substrates, leading to either loss of pharmacological activity or activation of prodrugs.^35^ The CYP3A family, particularly CYP3A4, is the predominant enzyme involved in phase I reactions, responsible for metabolising up to 60% of currently used drugs. This enzyme is primarily expressed in hepatic and intestinal tissues but is also found in the brain, prostate and stomach.^35^ CYP3A5, another significant enzyme in this family, is present in about 30% of individuals.^33,35^

CYP1A2, mostly expressed in the liver, accounts for a considerable portion of the hepatic CYP450 pool in some individuals.^33^ CYP2C9, also found in the liver, is the second most common CYP450 enzyme and is also present in the intestines and endothelial cells. Drugs metabolised by CYP2C9, such as warfarin, often have narrow therapeutic indices, requiring close monitoring in patients.^36–38^ Meanwhile, CYP2D6, primarily expressed outside the liver, metabolises approximately 25% of drugs from various therapeutic categories. In phase II reactions, non-CYP450 enzymes conjugate phase I products by adding acetyl, glucuronide, methyl or sulphate groups, producing typically inactive, polar by-products for renal or biliary excretion.^39^

### Diabetes-induced hepatic CYP450 modulation

Diabetes has long been associated with a state of low-grade inflammation, which also contributes to the disease’s progression.^40^ Research has linked pro-inflammatory cytokines with the modulation of CYP450 isoenzymes in both in vitro and in vivo studies.^41^ Given the pro-inflammatory state associated with diabetes, it is plausible to explore the modulatory effects of diabetes on CYP450 isoforms. Elevated levels of cytokines in patients with T2DM have been associated with changes in the expression and activity of certain CYP450 isoenzymes. Inflammatory conditions can impact the activity of transcription factors, which in turn affect the regulatory pathways of drug-metabolising enzymes.^42^ Most studies on this topic have been conducted in animals, where diabetes has been shown to modulate CYP450 enzymes. In STZ-induced T2DM models, significant reductions in CYP2C11 expression were observed, leading to decreased metabolism of drugs such as diclofenac, glibenclamide and nateglinide.^43,44^ Another study found that CYP1A2 was induced in STZ-diabetic rats, resulting in a lower area under the curve (AUC) for verapamil, with concurrent inhibition of intestinal CYP3A4 following both oral and intravenous administration.^45^

Although limited clinical studies have been conducted, diabetes has been shown to increase the activity of certain CYP450 isoforms.^46^ A recent study found that CYP2C19, CYP3A4 and CYP2B2 levels were elevated in diabetic patients, whilst CYP1A2 and CYP2C9 levels were slightly reduced.^46^ Since CYP450 activity plays a crucial role in determining the PK-PD response of several drugs, diabetes-induced alterations could lead to therapeutic challenges. For example, thrombosis is a common complication in diabetic patients, and reduced CYP2C19 activity may result in suboptimal efficacy of clopidogrel, a thrombolytic that relies on CYP2C19 for activation.^47^ Furthermore, CYP3A4 is involved in metabolising more than 40% of drugs, including many commonly prescribed for diabetes, making its modulation a significant concern for predicting drug response and adverse effects. Therefore, it remains elusive whether poor glycaemic control with some of the diabetic agents is associated with CYP450 modulation. The influence of diabetes on CYP450 modulation could be especially problematic for drugs with a narrow therapeutic index, where small differences between therapeutic and toxic doses can have serious consequences. For instance, reduced CYP2C19 activity may elevate plasma concentrations of drugs like voriconazole, an antifungal agent, potentially increasing the risk of adverse effects and complicating patient compliance.^48^ Given that diabetic patients are more susceptible to fungal infections, this is an important consideration.^49^

CYP450 modulation in diabetes has been strongly ascribed to inflammation, and to a lesser extent, obesity in T2DM patients.^47,50,51^ For example, no change in CYP2E1 activity was observed in non-obese T2DM patients, whilst increased activity was noted in obese patients. The study by Maximos et al. showed that the expression and activities of key CYP450s implicated in the metabolism of drugs were altered in diet-induced T2DM model.^52^ Interestingly, the observed effects were reversed when cells were exposed to CYP inducers. In the same study, it was shown that the mRNA levels of CYP3A11 and CYP3A25 in the liver were reduced by 2–14-fold, which correlated with a 21-fold reduction in midazolam metabolism.^52^ Dostalek et al., utilising human liver microsomes and midazolam as a probe, found that in samples from T2DM patients, CYP3A activity, mRNA and protein expression levels were decreased by 1.6–3.3-fold compared to those from non-diabetic patients.^53^ Although the precise mechanisms of inflammation-related CYP450 modulation remain unclear, changes at the level of gene expression have been observed, as evidenced by alterations in CYP450 mRNA expression. CYP450 enzymes are regulated at both transcriptional and post-transcriptional levels.^43^ Mechanistically, cytokines can affect transcription factors such as the nuclear receptor pregnane X receptor (PXR), as well as its dimerisation, thus, decreasing CYP450 mRNA expression.^43^ Other studies suggest that nuclear factor-kappa B (NF-κB) may regulate CYP450 expression by interacting with the promoter regions of CYP450 genes.^54,55^ Supporting this hypothesis, an in vitro study demonstrated that pro-inflammatory cytokines, including IL-1β, IL-4, IL-6, TNF-α and interferon gamma, suppressed CYP450 expression at the mRNA level in primary human hepatocytes.^56^

### Anti-hyperglycaemic-induced CYP450 modulation

Changes in CYP450 enzyme are not solely attributed to diabetes as a disease state, however, anti-hyperglycaemic agents have a modulatory effect on the CYP450 enzyme system. Recent in vitro studies have demonstrated that GLP-1 agonist has an inhibitory effect on CYP3A4 enzyme in cultured primary hepatocytes. Interestingly, a significant decrease in the CYP3A4 mRNA expression (57.2%–71.7%) and activity (18.5%–51.5%) were demonstrated with a clinically applicable concentration of 100 nM GLP-1 antagonist.^57^ Conversely, in a clinical setting these observations could not be replicated, therefore suggesting substantial variability between in vitro and in vivo systems.^57^

TZDs, including troglitazone, pioglitazone and rosiglitazone, have been shown to induce CYP3A4 in both in vitro and in vivo studies.^58^ On the other hand, TZDs have also demonstrated strong inhibition of CYP2C8 in vitro,^59^ suggesting the potential for DDIs at higher concentrations, especially for agents with narrow therapeutic indices. Thus, TZDs could increase the toxicity risk of CYP2C8 substrates. Using pooled human liver microsomes, Arnold et al. reported strong inhibition of CYP2C9 by rosiglitazone, resulting in reduced metabolism of warfarin.^60^ In a different study, a weaker inhibition of CYP3A4 was also observed.^61^ This finding indicates a potential for drug interactions with other CYP2C9-metabolised drugs, such as warfarin, diclofenac, amitriptyline, naproxen and phenytoin, potentially leading to toxicity and necessitating dose adjustments in diabetic patients on TZDs.

Although metformin is not commonly associated with significant DDIs, some studies suggest it could interact with other medications. Metformin has been shown to slightly reduce the PK parameters of aliskiren, with no expected PD change.^62^ Other studies have reported a decrease in the C_max_ and AUC of trospium but an increase in exposure to topiramate with metformin co-administration.^63,64^ These effects were not clearly linked to CYP450 modulation, as they were likely mediated by metformin interfering with the intestinal absorption of these drugs following oral administration. More recent reports, however, suggest that metformin can decrease CYP3A4 expression at the genetic level.^65^ This study found that metformin suppresses the PXR and other ligand-activated nuclear receptors involved in the regulation of CYP3A4. Considering that metformin can be co-administered with anticancer agents, this could have clinical implications as it may heighten the exposure to these agents through the inhibition of CYP3A4. Stanisławiak-Rudowicz et al. explored the bidirectional PK relationship between metformin and olaparib, a polyribose polymerase inhibitor used in BRCA-mutated cancer maintenance therapy. Unexpectedly, metformin did not alter the PKs of olaparib.^66^ Nevertheless, metformin as an adjuvant therapy in cancer should be monitored, as there is a potential for high exposure risks for some anticancer agents. It could be prudent to note that metformin benefits in cancers could be further ascribed to increasing exposure to anticancer drugs through the inhibition of CYP3A4.

## Plasma albumin

Albumin is the most abundant protein in plasma, accounting for nearly half of its total protein content, ranging from 3.5 to 5 g/dL in healthy individuals.^67^ Produced by hepatocytes, albumin is rapidly released into the systemic circulation at a rate of approximately 10–15 g per day. In humans, serum albumin serves several critical functions. It plays a key role in modulating plasma oncotic pressure, contributing to hemodynamic stability,^67,68^ and acts as a transporter of endogenous substances such as lipids and hormones.^69^ In the context of this paper, plasma albumin functions as a carrier and reservoir for various drugs, significantly impacting their PK and PD behaviours. The binding of drugs to albumin has been extensively studied, with two primary drug-binding sites located in the hydrophobic cavities of subdomains IIA and IIIA, known as Sudlow’s sites I and II, respectively.^70^ Clinically, serum albumin levels are determined through standard laboratory tests and are often used as an indicator of a patient’s nutritional status.^71^

Albumin concentration plays a pivotal role in the volume of distribution and clearance of highly protein-bound drugs, including antibiotics.^72^ Consequently, changes in plasma albumin concentration can affect drug PK-PD behaviour, as it serves as a reservoir. Albumin levels are often reduced in liver diseases and nephrotic syndrome due to decreased production and increased excretion, respectively.^73^ Clinical studies have shown that a reduction in albumin correlates with suboptimal therapeutic outcomes, particularly for highly protein-bound drugs that are time-dependent in their action. Examples of such drugs include ceftriaxone, ertapenem, flucloxacillin, teicoplanin, erythromycin, fusidic acid, oxacillin and daptomycin.

Jager et al. reported that in hypoalbuminaemic critically ill patients, flucloxacillin exhibited a significant increase in its volume of distribution, leading to lower drug concentrations than required for therapeutic efficacy.^74^ This suggests that higher doses of flucloxacillin may be necessary in patients with hypoalbuminaemia to achieve optimal therapeutic effects. Ramsey and MacGowan also reported a substantial decrease in the albumin binding of aztreonam in critically ill patients with hypoalbuminaemia compared to healthy subjects, resulting in increased total drug clearance.^75^ In renal impairment patients, Xu et al. observed similar increases in volume of distribution and clearance of aztreonam, leading to subtherapeutic antibacterial concentrations and compromised efficacy.^76^ Dhanani et al. further demonstrated that low plasma albumin levels were associated with reduced drug exposure and increased clearance of both total and unbound ertapenem and ceftriaxone.^77^ These findings are consistent with other studies that highlight the impact of hypoalbuminemia on the PKs of ceftriaxone. Collectively, these studies suggest that higher doses of highly protein-bound antimicrobials may be required to achieve therapeutic concentrations in patients with hypoalbuminaemia, ensuring an adequate antimicrobial effect.

### Diabetes and reduced albumin affinity

Diabetes has been linked to a reduction in plasma albumin concentration, potentially due to diminished hepatic synthetic function in the absence of insulin. Molecular studies indicate that insulin plays a critical role in preventing hypoalbuminaemia in diabetes by promoting albumin gene transcription in the liver through inhibition of Forkhead Box O1 (FOXO-1).^78^ In addition, high glucose levels can lead to the non-enzymatic glycation of albumin, altering its structure and biological function. This change impairs the binding affinity between albumin and drugs that rely heavily on albumin binding, thereby increasing the fraction of unbound drugs.^79,80^ For instance, the binding affinities of drugs such as warfarin and ketoprofen are reduced by albumin glycation, leading to altered efficacy in advanced diabetic patients.^81^

Ter Heine et al. demonstrated that phenytoin’s protein binding is influenced not only by serum albumin levels and the presence of severe renal impairment but also varies depending on its concentration. This may have clinical implications, as it could increase free phenytoin serum concentrations in diabetic patients.^82^ This alteration has significant clinical implications, particularly for drugs like warfarin, where even small changes in binding can result in major fluctuations in efficacy or toxicity. Sobczak et al. reported that dysregulated glucose levels have widespread effects throughout the body. Since fat and carbohydrate metabolism is closely linked, changes in plasma free fatty acid (FFA) levels and their metabolism are both a cause^2,3^ and an outcome^4^ of insulin resistance and type T2DM. Elevated FFAs contribute to a range of harmful effects, including chronic inflammation, pancreatic β-cell loss, atherosclerosis and heart disease, either triggering or worsening these conditions.^83^

In patients with T2DM, aspirin’s effectiveness depends on albumin levels. Hypoalbuminaemia is linked to reduced TxB2 inhibition and a higher risk of long-term cardiovascular events as reported by Sciacqua et al.^84^

Furthermore, glycation has been shown to alter albumin’s affinity for several sulphonylurea drugs used in treating T2DM, such as acetohexamide, tolbutamide and gliclazide (GLICL).^85^ This may lead to a higher concentration of the free drug in the systemic circulation, resulting in increased pharmacological activity and potentially toxic effects in diabetic patients. Żurawska-Płaksej et al. found that GLICL binds less strongly to glycated bovine serum albumin (BSA) compared to native BSA, which may result in a higher concentration of unbound drug in the systemic circulation.^86^ Recent studies have also demonstrated that albumin glycation reduces binding affinity for liraglutide, a GLP-1 agonist, possibly explaining the need for higher liraglutide doses in advanced diabetes cases to maintain efficacy.^87^

From all these studies, we can conclude that changes in plasma albumin could have clinical relevance as it may change the PK-PD of drugs in patients with diabetes resulting in sup-optimal treatment regimen and adverse effects. From the published studies, it is therefore prudent to consider routine assessment of plasma albumin concentration. Furthermore, clinicians should assess the risk of hypalbuminaemia in diabetics when managing disorders with highly protein-bound drugs, as it may necessitate exploring alternative options.

## Renal clearance

Renal excretion completes the drug elimination process, which often begins in the liver, although some drugs are excreted unchanged. Hydrophilic drugs, such as metformin, or their metabolites are filtered by the kidneys and do not undergo reabsorption.^88^ The urinary pH plays a crucial role in drug excretion, as the ionisation of drugs depends on the pH level. Renal disorders, such as chronic kidney disease (CKD), can impair this function and hinder drug excretion.^89^ As kidney function declines with age, drug excretion becomes less efficient, requiring dose adjustments. Beyond direct renal dysfunction, conditions that affect renal blood flow or urine flow can also influence drug elimination. Diabetes is a significant contributor to kidney failure and the increasing prevalence of CKD.

### DN and altered drug clearance

In diabetes mellitus (DM), the early phase of kidney dysfunction is characterised by a supraphysiological rise in the GFR.^90^ This clinical condition, known as glomerular hyperfiltration, results from structural and dynamic changes that regulate GFR.^90^ Over time, uncontrolled hyperglycaemia causes a decline in GFR due to glomerular basement membrane thickening, driven by mesangial expansion.

DN is one of the major microvascular complications of DM, often culminates in CKD and end-stage renal disease (ESRD).^91–94^ DN is typically defined by macroalbuminuria (>300 mg in 24 h) or by microalbuminuria alongside impaired renal function, indicated by abnormalities in creatinine clearance or GFR and serum creatinine levels. Approximately one-third of diabetic patients develop DN. The PK properties of drugs primarily excreted by the kidneys are altered due to impaired renal function,^95^ with changes in GFR affecting patients with type 1 diabetes and, potentially, T2DM. These alterations can influence the PD profiles and safety of the drugs, often necessitating dose adjustments. Insulin and metformin are two antidiabetic agents whose clearance occurs primarily through the kidneys. DN has been shown to delay insulin clearance, leading to accumulation and hypoglycaemia.^88,96^ A retrospective cohort study involving approximately 244,000 patients, both with and without diabetes, revealed that patients with CKD experienced a higher frequency of hypoglycaemic events compared to those without CKD.^97^ Similarly, a study by Do et al reported that hospitalised patients with diabetes, CKD and ESRD are linked to a higher risk of hypoglycaemia.^98^ Therefore, it can be concluded that severe hypoglycaemia in the presence of diabetic kidney disease increases the risk of mortality.^99^ Prolonged hypoglycaemia can also lead to complications such as brain damage, arrhythmias and coma.^100^ Regarding metformin, poor renal clearance leads to its accumulation, which can increase the risk of lactic acidosis.^101^

A study by Sarashina et al. indicated that renal function loss is likely to alter the excretion of empagliflozin, though its metabolism remained unchanged.^102^ Empagliflozin’s mechanism of action depends on the filtered glucose load in the glomeruli, which is regulated by the GFR.^103^ The increase in urinary glucose excretion is inversely proportional to the intensity of renal impairment in diabetic patients. It is well established that long-term uncontrolled diabetes can lead to both micro- and macrovascular complications, which may result in an increased GFR.^93^ Some researchers have investigated how diabetes affects GFR when using antibiotics. Tran reported on numerous studies, one study found a significant increase in GFR and the clearance of penicillin G in diabetic children compared to healthy subjects of the same age.^104^ However, the clearance and serum concentrations of renally excreted drugs such as kanamycin, benkanamicin and amikacin were found to be similar in both diabetic and non-diabetic patients.^104^ Given these varying results, it is challenging to predict the impact of diabetes on renal function and the renal clearance of drugs.

There are few studies that focus on DN-related alterations in drug PDs, as compared to changes in PKs. Due to varying findings, the impact of DN on the PKs of various drugs remains unclear, underscoring the need for more clinical studies to establish the clinical relevance of these PK changes.^105^ It remains challenging to determine whether current studies accurately reflect both PD and PK alterations. Improper dosing in patients with CKD can result in either toxicity or ineffective therapy, with older patients being particularly vulnerable due to age-related declines in renal function and polypharmacy for comorbid conditions.^106^ CKD affects multiple aspects of drug metabolism, including glomerular blood flow, filtration, tubular secretion, reabsorption, renal bioactivation and metabolism.^107^ Furthermore, drug absorption, bioavailability, protein binding, volume of distribution and non-renal clearance may also be altered in these patients.^108^ Clinicians must be cautious when prescribing drugs with active or toxic metabolites that could accumulate, exacerbating pharmacological effects or adverse reactions in CKD patients.^107^

**Figure 1.**
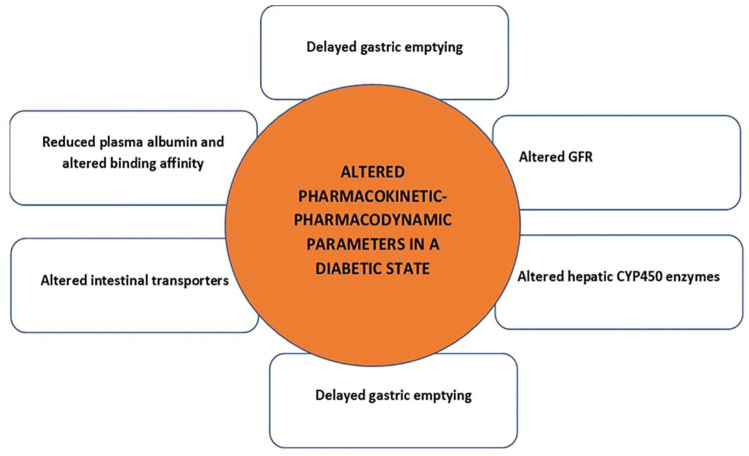
The PK-PD profile of drugs is prone to alterations due to delayed gastric emptying, changes in intestinal drug transport, hepatic CYP450 enzymes, plasma albumin concentrations and drug-binding affinity, GFR. CYP450, cytochrome P450; GFR, glomerular filtration rate; PK-PD, pharmacokinetic-pharmacodynamic.

Many antimicrobial agents require dosage adjustments in CKD patients due to their renal elimination, although some commonly used antibiotics do not require such adjustments.^109^ Elevated serum levels of penicillin G, for example, can lead to adverse reactions such as neuromuscular toxicity, seizures or coma.^110^ Imipenem/cilastatin may accumulate in CKD, potentially causing seizures if doses are not reduced.^111^ In the advanced stages of the disease, alternative carbapenems like meropenem should be considered.^112^ Nephrotoxicity from tetracyclines is most commonly associated with proximal tubule damage, leading to Fanconi syndrome, which typically occurs after the use of degraded or outdated tetracyclines. Less frequently, acute interstitial nephritis has been reported in connection with minocycline and doxycycline.^113^ Studies have shown that nitrofurantoin can accumulate in CKD patients, leading to toxic metabolites and causing peripheral neuropathy.^114^ Aminoglycosides should be avoided in CKD patients when possible.^115^ When initiating treatment, initial doses should be based on accurate GFR estimations, with close monitoring of renal function and drug concentrations to guide further dosage adjustments. Consequently, the administration of antibiotics in CKD patients may lead to suboptimal management of the condition, as the pharmacological agent may accumulate within the patient’s system, exacerbating adverse antimicrobial effects. This, in turn, can lead to improper patient management. In Figure 1, we have provided factors influencing PK-PD changes in a diabetic state.

## Evidence of altered state PK-PD of some examples of pharmacological agents in T2DM

The PKs of various drugs has been studied in both animal models of diabetes and clinical settings, though conflicting observations have been reported. In this section, we highlight the changes in PK and PD profiles of drugs used to treat common diseases in T2DM, such as infectious and cardiovascular diseases. We will briefly explore recent experimental and clinical findings.

In a clinical study, the PKs of antituberculosis drugs were examined in both diabetic and non-diabetic patients during the intensive therapy phase.^116^ However, no significant differences were observed between the groups regarding PK parameters. A study conducted 5 years later reported delayed rifampicin absorption, along with increased renal clearance in T2DM patients compared to healthy subjects.^117^ DM can also impede drug distribution into tissues due to microvascular changes and reduced vascular permeability. Given the high incidence and severity of diabetic foot infections, and the potential for reduced tissue distribution, understanding antimicrobial tissue penetration in DM patients is essential, particularly in diabetic foot infections. Vancomycin concentrations in diabetic soft tissues, for example, have been found to be lower compared to non-diabetic tissues.^118^ However, conflicting reports exist, with one study showing no significant changes in vancomycin trough concentrations.^119^ These variations may be attributed to differences in analytical methods and sample sizes.

Candidiasis, a common fungal infection in diabetes, presents treatment challenges due to altered PK profiles. Many antifungal drugs, such as amphotericin B, itraconazole, ketoconazole, miconazole, caspofungin, anidulafungin and micafungin, are highly protein-bound.^119^ A recent study has demonstrated that, antifungal binding was found to be reduced in diabetic patients, whilst fluconazole binding remained unaffected.^120^ An observational study explored the relationship between the AUC to minimum inhibitory concentration ratio of fluconazole and clinical outcomes in patients with candidemia. The study found that higher doses of fluconazole were necessary for effective Candida treatment.^121^

Cardiovascular diseases are more common in diabetes than in non-diabetic individuals, increasing the probability that diabetic patients will require cardiovascular agents. The diabetic state may enhance the effects of digoxin, as studies have shown that digoxin clearance is significantly reduced in patients with type 2 diabetes compared to healthy controls.^122^ Verapamil, on the other hand, has demonstrated opposing PK effects, with increased plasma concentrations following oral administration and decreased levels after intravenous administration. These findings concluded that diabetes may accelerate the metabolism of verapamil in rats, and this increased metabolism could be partially attributed to the induction of CYP3A enzymes.^123^ In a separate study, it was observed that calcium channel blocker (CCB) treatment was found to be negatively correlated with HbA1c levels and positively associated with β-cell function in hypertensive patients with T2DM. This suggests that CCBs may be a suitable treatment option for hypertensive T2DM patients with diminished β-cell function.^124^

The efficacy of anticoagulants is also altered in diabetic patients. A recent study demonstrated that warfarin binds more strongly to albumin in a diabetic state, reducing the unbound fraction in plasma. This finding is consistent with earlier studies that reported reduced warfarin efficacy in diabetic patients.^125^ Interestingly, no changes were observed in bound heparin levels in the same study. Through potential challenges associated with non-direct coagulants, direct anticoagulants are becoming increasingly popular in the management of diabetic patients with cardiovascular diseases due to their extensive evaluation in large phase III trials, demonstrating efficacy and safety comparable to, or better than, those in non-diabetic patients.^126^

Cholesterol-lowering agents are frequently used in diabetic patients to improve cardiovascular outcomes. Evidence from a recent study suggests that oral exposure to atorvastatin increased in a diabetic animal model due to decreased intestinal OATP1B2 and hepatic CYP3A expression.^126^ In contrast, simvastatin plasma concentrations were found to be decreased in diabetic rats, correlating with increased OATP1B2 and CYP3A expression at the mRNA level.^127^ Parting this section, we will explore loop-diuretics PK parameters, as they have shown variable PK effects in diabetes. Torsemide has been associated with a high risk of hyperglycaemia and increased drug clearance, while conflicting results were observed in alloxan-induced diabetic rats, suggesting that urinary flow rate may be a factor.^128^ A recent clinical study reported that diabetic patients with acute heart failure were more inclined to require intravenous diuretic therapy. Furthermore, such patients were more frequently discharged with higher doses of furosemide.^129^ Although such probable PK-PD changes could be manifested in diabetes due to pathophysiological mechanisms associated with the disease; however, interventions aiming to control diabetes envisaged to revert such changes. In the preceding section, we are going to example few examples of conventional and newer anti-hyperglycaemic on their effect and examples of drug interactions they have.

## The effect of conventional DM therapies

Understanding the effects of T2DM as a disease on certain body mechanisms and the effects of drugs used to manage it are crucial for effective treatment and management. The conventional pharmacotherapy for diabetes includes insulin, metformin and sulphonylureas (Table 1). The tight glycaemic control afforded through these agents is critical in prevention and delaying the onset and progression of pathophysiological states described above, which can alter PK-PD. This, therefore, further highlights the importance of glycaemic control in diabetic patients. Through innovations in diabetes drug discovery and development, several new therapies for T2DM have emerged in recent years, offering improved blood glucose control with added benefits such as weight loss and cardiovascular protection. The newer agents include DPP4 inhibitors, SGLT2 inhibitors and GLP-1 receptor agonists (Table 1) These advancements are particularly beneficial in personalised diabetes management, helping to address both glycaemic control and associated complications of T2DM such as cardiovascular and renal diseases. In Table 1 we have briefly explored the commonly utilised conventional and newer pharmacotherapies, looking and their disease-modifying effect and possible alteration of PK-PD of other drugs.

**Table 1.** Examples of conventional and new diabetes therapies and their pharmacotherapeutic effect and possible alteration of PK-PD of some drugs.

Drug examples	Effect on the disease	Effect of PK-PD on other drugs	Metabolism and interactions	References
Insulin therapy	Insulin is known to afford glycaemic control thus delaying the onset and progression diabetes complications. Over the years, insulin analogues with varied PK-PD profiles have been developed with a goal to closely match physiological insulin secretion. These analogues have revolutionised the management of diabetes.	Insulin does not interact with drugs through the traditional CYP450 mechanism. However, various drugs are known to alter the PD response to insulin.	Insulin metabolism is a receptor-mediated process. Once insulin binds to its receptor, it functions as a carrier, facilitating the delivery of insulin to the bloodstream and intracellular compartments. Inside the cell, the delivered insulin is located in the cytosol, Golgi apparatus and nucleus. Insulin interacts with various medications, including other diabetes treatments, blood pressure drugs and atypical antipsychotics. Corticosteroids, certain antibiotics and alcohol can also affect insulin’s action. These interactions frequently lead to low blood glucose levels.	130–133
Sulphonylureas	Sulphonylureas stimulate insulin secretion, thus affording glycaemic control.	Tolbutamide has been shown to increase serum concentration of abrocitinib and acamprosate.	Sulfonylureas are extensively metabolised in the liver by cytochrome CYP2C9 isoform, and hence patients on medications that inhibit or activate this enzyme may be subjected to sulphonylurea toxicity or impaired metabolism. The azole antifungals such as fluconazole are known inhibitors of CYP2C9 activity, co-administration with glimepiride can prolong the elimination phase half-life and increase the maximum plasma concentration of glimepiride leading to sulphonylurea toxicity.	134–137
Metformin	Metformin, a commonly used oral medication for T2DM, improves insulin sensitivity in muscle cells and decreases glucose production in the liver. This helps lower blood glucose levels by making the body’s cells more responsive to insulin.	Metformin has been reported to increase the serum concentrations of the following drugs: aclidinium, acrivastine, acyclovir, abacavir, abemaciclib.	The oral absorption and hepatic uptake of metformin are mediated possibly by OCTs (OCT1 and OCT2), therefore, varied therapeutic efficacy was observed when various drugs that interact with OCTs were co-administered with metformin. Dolutegravir, being an inhibitor of both OCT2 and MATE1 transporters within the renal tubules; when co-administered with metformin may increase the risk of hypoglycaemia and GI intolerance due to increased plasma concentrations of metformin, thus requiring dose adjustment.	137–143
DPP4 inhibitors (sitagliptin)	DPP4 inhibitors extend the half-life of incretins, thus promoting insulin secretion. DPP4 inhibitors’ benefits have been shown to extend beyond glycaemic control, for example improving kidney and cardiovascular function.	Sitagliptin has not been reported to interact with many drugs. However, it can increase serum concentration of aclidinium.	85% of the absorbed dose of linagliptin is eliminated in faeces via biliary excretion which does not significantly impact CYP450 metabolism, making it less likely to interfere with other medications. However, saxagliptin necessitates dose adjustment if administered concurrently with medications that strongly inhibit CYP3A4. Saxagliptin is metabolised to its active form by CYP3A4, hence the level of this drug and its active metabolite might be enhanced when co-administered with drugs affecting this isoenzyme such as ketoconazole (CYP3A4 inhibitor) and rifampicin (CYP3A4 Inducer).	144–147
SGLT2 inhibitors	SGLT2 inhibitors have been shown to reduce the risk of major adverse cardiovascular events in patients with T2DM. These drugs promote glucose excretion in urine, which not only helps control blood glucose levels but also has beneficial effects on blood pressure and weight, reducing cardiovascular risk. These drugs can lead to modest reductions in blood pressure due to their diuretic effect, which helps alleviate fluid overload and reduce cardiovascular stress. Furthermore, reno-protective effect has been reported.	Canagliflozin has been shown to increase digoxin exposure. Dapagliflozin has been shown to modestly increase serum simvastatin concentration.	Primarily eliminated unchanged through the kidneys. However, SGLT2 inhibitors undergo biotransformation by UGT mediated glucuronidation. UGT enzyme inducers, such as rifampin, phenytoin and ritonavir, reduce canagliflozin exposure which may reduce the effectiveness of canagliflozin. The dose of canagliflozin when used with UGT enzyme inducers should be increased.	148–152
GLP-1 receptor agonists	GLP-1 receptor agonists potentiate insulin secretion and further assist with weight management by enhancing satiety and reducing appetite. They slow gastric emptying.	The slowing effect of gastric emptying might change oral drug absorption, potentially affecting PKs. Nevertheless, no significant drug-interaction have been reported. Moreover, more studies are required to further consolidate the effects of GLP-1 receptor agonists.	GLP-1 is rapidly metabolised and inactivated by the enzyme dipeptidyl peptidase IV, often before it leaves the gut. This suggests that GLP-1’s effects may be mediated through sensory neurons in the intestine and liver that express the GLP-1 receptor, rather than through systemic circulation. PK-PD studies of GLP-1 receptor agonists typically indicate that there are no clinically significant alterations in the absorption or efficacy of oral medications taken concurrently. Therefore, dose adjustments are generally unnecessary when using GLP-1 receptor agonists alongside oral medications. However, it is advisable to exercise caution when treating patients with pre-existing gastroparesis or kidney dysfunction, or when prescribing drugs that have a narrow therapeutic index.	153,154

CYP450, cytochrome P450; OCT, organic cation transporter; PD, pharmacodynamic; PK, pharmacokinetic; T2DM, type 2 diabetes mellitus; UGT, uridine 5′-diphosphate-glucuronosyltransferases.

## Author perspective and conclusions

T2DM is associated with changes in the renal function, from higher GFR during the onset of the disease and decline in GFR in overt cases, thus altering PK-PD behaviour of numerous drugs therefore altering their efficacy.

From the above literature, we have associated diabetes with delayed gastric emptying and intestinal drug transporters changes, a reduction in plasma albumin as well as the hepatic CYP450 enzyme system dysregulation in part through increases in the inflammatory state. T2DM therefore presents a special case, where there is a necessity for continuous monitoring of drug PK-PD alterations especially for drugs with lower therapeutic index and highly fatal infectious diseases. New drugs are being used in combination therapies to maximise benefits, offering synergistic effects on weight, glucose control and cardiovascular health. These advancements are particularly beneficial in personalised diabetes management, helping to address both glucose control and associated complications like cardiovascular and renal diseases.

More studies aiming to understand and predict PK-PD changes in DM using appropriate models that mimic the pathophysiology state of diabetes are encouraged otherwise these observations cannot be replicated in clinical diabetes. Through these studies, efficacious intervention whilst minimising adverse effects could be sought. More importantly, upcoming clinical trials comprised of multidisciplinary experts should strive to assess the impact of diabetes on a particular drug elimination pathway and underlying mechanisms. These observations should not be generalised but consider the existence of comorbid disease states where possible, as the physiology conditions may vary, therefore showing different PK-PD outcomes. Most importantly, effective diabetes management and monitoring should be intensified to prevent diabetic pathological conditions that could affect PK-PD properties of other agents for co-morbidities.

## Declarations

**Ethics approval and consent to participate:** Not applicable.

**Consent for publication:** All authors have consented for the publication.

**Availability of data and materials:** Not applicable.
